# Mortality trends and risk factors in advanced stage-2 Human African Trypanosomiasis: A critical appraisal of 23 years of experience in the Democratic Republic of Congo

**DOI:** 10.1371/journal.pntd.0006504

**Published:** 2018-06-13

**Authors:** Léon Mbiyangandu Kazumba, Jean-Claude Tshinzobe Kaka, Dieudonné Mumba Ngoyi, Désiré Tshala-Katumbay

**Affiliations:** 1 Départment de Neurologie, Université de Kinshasa, Kinshasa, République Démocratique du Congo (RDC); 2 Institut Supérieur de Techniques Médicales, Kinshasa, Démocratique du Congo (RDC); 3 Départment de Médecine Tropicale, Université de Kinshasa, Kinshasa, Démocratique du Congo (RDC); 4 Institut National de Recherches Biomédicales (INRB), Kinshasa, Démocratique du Congo (RDC); 5 Départment of Neurology and School of Public Health, Oregon Health & Science University, Portland, OR, United States of America; Institute of Tropical Medicine, BELGIUM

## Abstract

We conducted a retrospective study on mortality trends and risk factors in 781 naïve cases of advanced stage-2 sleeping sickness admitted between 1989 and 2012 at the National Reference Center for Human African Trypanosomiasis (HAT), Department of Neurology, Kinshasa University, Democratic Republic of Congo (DRC). Death was the outcome variable whereas age, gender, duration of disease, location of trypanosomes in body fluids, cytorachy, protidorachy, clinical status (assessed on a syndromic and functional basis) on admission, and treatment regimen were predictors in logistic regression models run at the 0.05 significance level. Death proportions were 17.2% in the standard melarsoprol schedule (3-series of intravenous melarsoprol on 3 successive days at 3.6 mg/kg/d, with a one-week interval between the series, ARS 9); 12.1% in the short schedule melarsoprol (10 consecutive days of intravenous melarsoprol at 2.2 mg/kg/d, ARS 10), 5.4% in the first-line eflornithine (14 days of eflornithine at 400 mg/kg/d in 4 infusions a day DFMO B), 9.1% in the NECT treatment regimen (eflornithine for 7 days at 400, mg/kg/d in 2 infusions a day combined with oral nifurtimox for 10 days at 15 mg/kg/d in 3 doses a day); and high (36%) in the group with select severely affected patients given eflornithine because of their clinical status on admission, at the time when this expensive drug was kept for treatment of relapses (14 days at 400 mg/kg/d in 4 infusions a day, DFMO A). After adjusting for treatment, death odds ratios were as follows: **10.40** [(95% CI: 6.55–16.51); p = .000] for clinical dysfunction (severely impaired clinical status) on admission, **2.14** [(95% CI: 1.35–3.39); p = .001] for high protidorachy, **1.99** [(95% CI: 1.18–3.37); p = .010] for the presence of parasites in the CSF and **1.70** [(95% CI: 1.03–2.81); p = .038] for high cytorachy. A multivariable analysis within treatment groups retained clinical status on admission (in ARS 9, ARS 10 and DFMO B groups) and high protidorachy (in ARS 10 and DFMO B groups) as significant predictors of death. The algorithm for initial clinical status assessment used in the present study may serve as the basis for further development of standardized assessment tools relevant to the clinical management of HAT and information exchange in epidemiological reports.

## Author summary

This study reports on the importance of recording data related to the clinical presentation on admission in sleeping sickness. We collected data on 781 second-stage patients admitted in the Trypanosomiasis Unit of the Department of Neurology of the University of Kinshasa from 1989 to 2012. The following information were registered: age, sex, symptomatology duration, trypanosome parasites location in body fluids, clinical presentation on admission, cerebrospinal fluid (CSF) cell count, CSF protein concentration, therapeutic regimen, and post-treatment outcome in terms of death or survival. Analyses demonstrated that the most influential factor on the occurrence of death was a dysfunctional clinical status on admission (reflecting advanced disease). These results support the idea that the algorithm used to categorize patients in the present work can be proposed for validation in prospective studies with the purpose of designing a standardized clinical procedure assessing disease severity and bearing predictive value of outcome.

## Introduction

The clinical management of HAT has always been confronted with the issue of treatment efficacy, drug toxicity and mortality risk factors [1–6]. In the process of assigning the probable cause of death to disease progression, treatment side effects, or any other factors including but not limited to coexistent pathology, the assessment of clinical status on admission among the baseline characteristics may be crucial [2,7]. The clinical presentation of HAT has been abundantly described in previous literature [8–12]. After a period of general manifestations (Stage 1), the gambiense form of HAT is characterized by a protracted course with a vast spectrum of symptoms and signs reflecting progressive involvement of multiple sites of central and peripheral nervous system (CNS, PNS) (Stage 2) that can last several years. The heterogeneous symptomatology that ensues can be summarized in three main categories:alertness and mental disturbances, motor and sensory symptoms, and sleep disturbances that owed the disease its name. If untreated the patient enters a terminal stage with incoherence, repeated seizures, double incontinence, eventually cachexia, coma and death, as a result of major brain demyelination and atrophy [13].

The pertinence of including clinical aspects in the current case definition has been perceived by experts who called for standardization of HAT clinical records [7,14–17] However some authors have raised concerns about the interpretation of clinical data collected by examiners with different level of training [2,11,12] and only few studies have addressed the issue of the prognostic value of clinical presentation in treated HAT [9,10,18,19]. With the advent of new diagnostic tools and safe, easy-to-take medicines, the WHO has planned to maintain the present decreasing endemicity trend and considers elimination of HAT in the coming years a realistic target [20–22]. Some long-standing practices such as the current mandatory requirement of accurate stage determination for drug allocation are likely to be revised soon [20–22]. One of the strategic changes in the fight against HAT is a special emphasis on passive case detection further involving fixed health care settings and launching new insights on the clinical aspects of the disease. It is therefore of paramount relevance that HAT health-care workers and scientists are provided with a standardized clinical evaluation tool. Such assessment tool would facilitate comparison of results in large scale multicenter studies.

The present paper draws on a 23-year-experience of care to sleeping sickness patients in a specialized center and intends to assess the impact of pre-treatment clinical status as well as select biological factors on mortality rate.

## Methods

We retrospectively examined hospital records of naïve cases of stage-II HAT admitted and treated in the Unit of HAT of the Department of Neurology at the University of Kinshasa from 1989 to 2012. The aforementioned unit was officially designated in March 2001 to serve as the National Reference Centre for HAT in collaboration with the Congo National Program against HAT. Sleeping sickness diagnosis is completed at the CNRTHA or through the referral system of the National Program (Mobile teams or select first-level medical centers). Ethical approval was obtained from the institutional Review Board of the INRB (Institut National de Recherches Biomédicales) and all data analyzed in our study were anonymized to protect confidentiality.

### Inclusion and exclusion criteria

Subjects with confirmed parasitological diagnosis of HAT (trypanosomes detected by direct microscopic examination of blood, lymph juice or cerebrospinal fluid), stage-2 disease, and complete medical records were included in the present study. Subjects were considered in stage-2 HAT when trypanosomes were found in the CSF and, or the white blood cell (WBC) count in the cerebrospinal fluid (CSF) was more than 5 cells/m^3^ and / or the total protein concentration > 45 mg/dl (Biuret method) [23,24]. This latter exam is routinely practiced at the CNRTHA, but is not part of the current investigations in first-level settings. Cases in stage-1 disease or admitted for sequelae or relapse were not included in the study (S1 **Diagram Flow**).

### Variables of interest

Death was considered as the dependent variable. Independent variables (predictors) included age, sex, duration of the disease, location of the parasites in body fluids at the time of the diagnosis, CSF WBC, CSF total protein concentration, clinical status on admission, and treatment regimen.

The clinical status on admission was categorized following a bi-axial clinical assessment inventory set up on the ground of our experience in the management of sleeping sickness patients. This assessment procedure is similar to that reported in a previous study [19]. In the new version we added to the assessment based on the record of clinical signs (syndromic axis) an evaluation of the functioning based on the level of impairment of three basic functions: speaking, walking and swallowing (functional axis). For each patient the following data were extracted from the medical file. In one hand the presence or absence of nine clinical items: behavioral changes or altered awareness (any modification of any aspect of behavior), archaic reflexes (palmo-mental reflex or grasping reflex), rigidity (cog-wheel or lead-pipe hypertonia), cerebellar dysfunction (intention tremor or dysmetria or dysdiadochokinesia or wide-based gait), dyskinesia (chorea or athetosis or dystonia), hemiparesis, incontinence, neck stiffness, and weight loss); in the other hand the level of impairment in speaking, walking and swallowing quoted as intact, partially impaired or lost, The assessment procedure is shown in the chart below (**Table 1**).

**Table 1:** Stage-2 HAT severity assessment chart.

Data from clinical examination on admission		Severity assessment	
**Number of signs present (out of 9)**	**Level of impairment of 3 functions**	**Severity grade**	**Dichotomized category**
Absent (Asymptomatic)	All functions preserved	Grade 0	Non-Dysfunctional
1 to 2 signs (Paucisymptomatic)	Partial impairment of 1 or 2 functions	Grade 1	Non-Dysfunctional
3 or more signs (Multisymptomatic)	Partial impairment of 1 or 2 functions	Grade 2	Non-Dysfunctional
Any number of signs	Partial impairment of 3 or complete loss of 1 or 2 functions = “hypofunctional status”)	Grade 3	Dysfunctional
Any number of signs	All functions lost (“afunctional status” = akinetic mutism)	Grade 4	Dysfunctional

Disease severity is assessed as “Non-Dysfunctional” (Grades 0, 1 or 2) or “Dysfunctional” (Grades 3 or 4).

Patients were treated according to the guidelines of the WHO and the National HAT Program. The following regimens were used over time: 3 series of intravenous (i.v) melarsoprol on 3 successive days at 3,6 mg/kg/d, with a one-week free interval between the series (long schedule melarsoprol regimen, herein referred to as ARS 9) from 1989 to 2000; 10 consecutive days i.v. melarsoprol at 2.2 mg/kg/d (short schedule melarsoprol regimen, herein referred to as ARS 10) from 2000 to 2006; 14 days of eflornithine at 400 mg/kg/d in 4 infusions a day (first-line eflornithine regimen, herein referred to as DFMO B) from 2007 to 2009; a combination of eflornithine for 7 days at 400 mg/kg/d in 2 infusions a day with oral nifurtimox for 10 days at 15 mg/kg/d in 3 doses a day (herein referred to as NECT regimen) from 2010 to 2012. Eflornithine was also given at the same dosage as DFMO B to select severely affected patients to avoid exposing them to the risk of melasoprol severe adverse events (selective eflornithine, herein referred to as DFMO A regimen) between1989 and 2006. Patients with deglutition difficulties were fed or given drugs *via* a nasogastric tube.

### Statistical analysis

Data on age, duration of disease, WBC, and total protein concentration in the CSF were dichotomized using arbitrary cut-off points of 15 years, 6 months, 100 WBC/ml, and 100 mg protein/dl, respectively. Clinical status on admission was dichotomized into “dysfunctional status” and “non-dysfunctional status” and parasitological diagnosis was analyzed considering the presence of trypanosomes whether in CSF or not. Treatment regimens were compared in reference to DFMO B regimen. Logistic regression models were used to assess the association between death and the aforementioned independent variables including gender. Statistical analyses were conducted using Stata software (version 11.2, Stata Corp Inc.) at the significance level of 0.05.

## Results

### Yearly admission rates of the 781Stage-2 HAT patients

**Fig 1:**
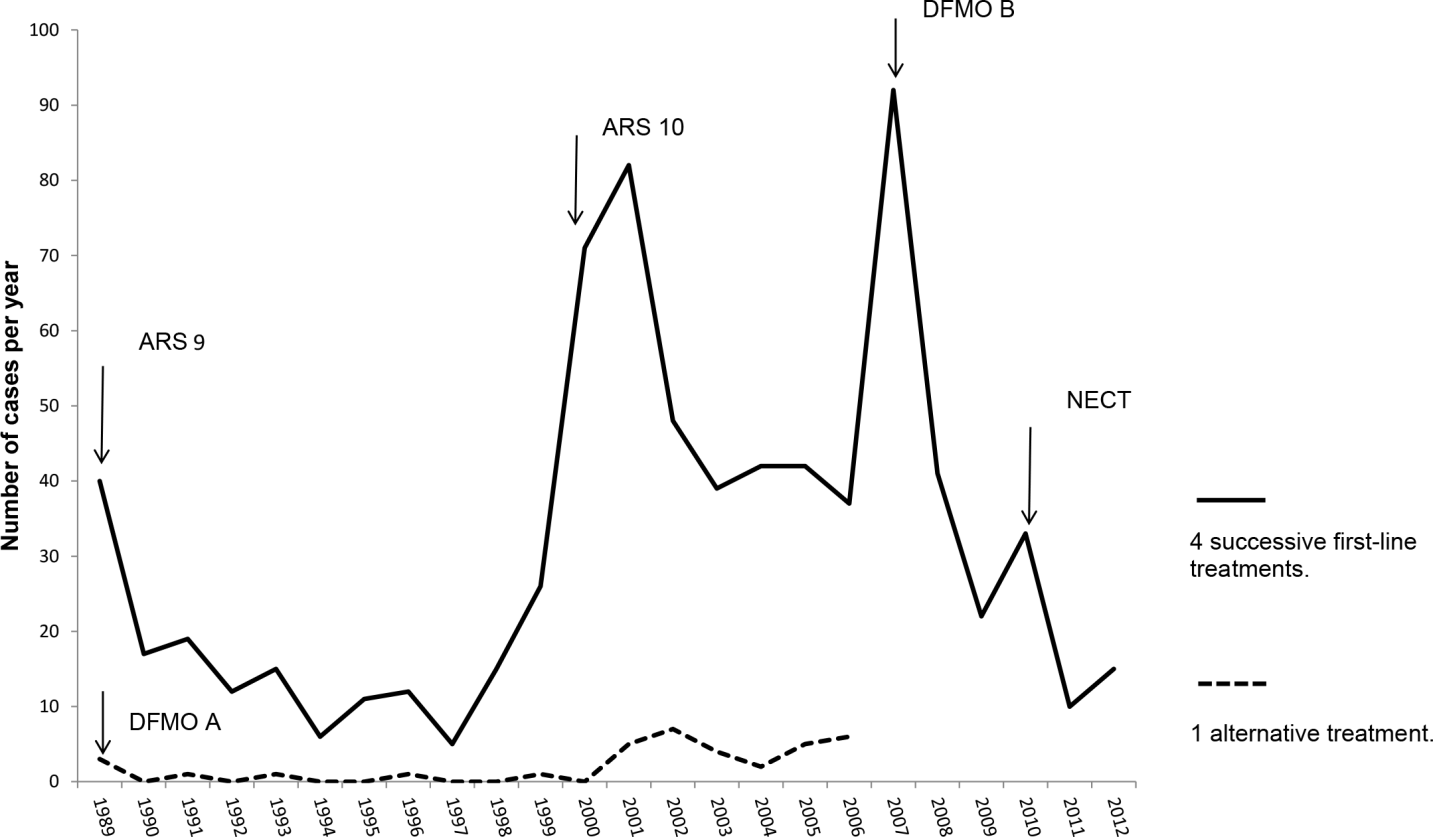
Yearly admissions of study subjects and treatment regimens over the study period. Arrows indicate introduction of select treatment regimens. Plain line = 4 successive regimens (ARS9 or melaroprol long schedule, ARS10 or short schedule melarsoprol, DFMO B or first-line eflornithine and NECT or nifurtimox-eflornithine combination therapy). Dotted line = period when severely affected patients were exceptionally treated with eflornithine (DFMO A), which was the preferred option for relapsed cases. Peaks in admissions correspond to the discovery of new foci of sleeping sickness in the villages of Kimwenza in 2001 and Muwana in 2007.

Admissions rates remarkably changed over the study period and were the highest in 2001 and 2007, mainly due to the discovery of new endemic foci of HAT in the villages of Kimwenza and Muwana, respectively. The decrease in the rate of admissions from 2009 and onwards followed the drawback of the HAT endemicity in the country (Fig 1).

### General and clinical characteristics in the 781 study subjects

The frequencies of clinical features from the syndromic and functional assessments are reported in **Tables 2 and 3**while the data on the variables of interest are displayed in **Table 4**.

**Table 2:** Syndromic and functional aspects of clinical presentation in the 781 study subjects.

Syndromic Assessment	N	%
Behavioral changes or Altered awareness	536	68.6
Archaic reflexes	466	60.1
Rigidity	428	54.8
Weight loss	428	54.8
Neck stiffness	380	48.7
Cerebellar signs	356	45.6
Incontinence	212	27.1
Hemiparesis	172	22.0
Dyskinesia	114	14.6
**Functional Assessment**		
Walking Impairment	207	26.5
Speech Impairment	186	23.8
Deglutition Impairment	91	11.6

**Table 3:** Frequency by severity grade in the 781 study subjects.

Severity grade	N	%
Grade 0	84	10.8
Grade 1	416	53.3
Grade 2	137	17.5
Sub-Total (Non-Dysfunctional)	**637**	**81.6**
Grade 3	101	12.9
Grade 4	43	5.5
Sub-Total (Dysfunctional)	**144**	**18.4**
Total	**781**	**100**

**Table 4:** Socio-demographic, biological and clinical characteristics by treatment group.

Characteristics		ARS 9, N = 226	ARS 10, N = 307	DFMO A, N = 36	DFMOB, N = 168	NECT, N = 44	OVERALL, N = 781
**Sex**	**Males**	133	152	14	88	26	413
	**Females**	93	155	22	80	18	368
	**Ratio M/F**	1.4	1	0.6	1.1	1.4	1.1
**Age (years)**	**Mean±SD**	30.7 ± 15.0	30.8 ± 16.5	23.1 ± 20.8	29.3 ± 17.3	29.5 ± 11.0	30.0 ± 16.3
	**% (<15 y)**	14.2	16.9	47.2	23.2	4.5	18.2
**Duration (months)** ^**a**^	**Mean± SD**	11.4 ± 13.6	9.0 ± 11.9	8.4 ± 5.4	11.1 ± 15.2	5.7 ± 9	9.9 ± 12.8
	**% (≥6 m)**	66.7	21.2	57.6	50.6	36.4	51.8
**Clinical presentation on admission**	**% (Dysfunctional)**	17.3	13.4	66.7	13.7	18.2	18.4
**Parasites location**	**% (CSF+)** ^**b**^	75.1	66.8	83.3	59.5	90.9	69.9
**CSF WBC (count/ml)** ^**c**^	**Mean± SD**	256.2 ± 303	397.9 ± 456	463.4 ± 489.3	347.0 ± 478.6	473.7 ± 382.6	353.3 ± 425.4
	**% (≥100)**	67.9	70.0	86.1	66.1	90.7	70.4
**CSF Proteins (mg/dl)**	**Mean± SD (N)**	116.6 ± 56.3 (207)	89.4 ± 35.6 (217)	108.5 ± 56.4 (28)	85.6 ± 24.8 (75)	88.4 ± 13.7 (22)	99.8 ± 45.7 (559)
	**% (≥100)**	57.9	29.0	35.7	17.3	11.4	37.7
**CSF WBC ≤ 5** ^**d**^	**N**	15	4	1		1	21

SD = standard deviation; CSF = cerebrospinal fluid; WBC = white blood cells.

^a^ Data were lacking in 22 subjects

^b^ Percentage of cases with trypanosomes present in their CSF

^c^ Data were lacking in 3 subjects

^d^ Subjects classified as stage 2 on the basis of high CSF proteins (≥ 100 mg/dl).

### Death rates and risk factors

**Fig 2:**
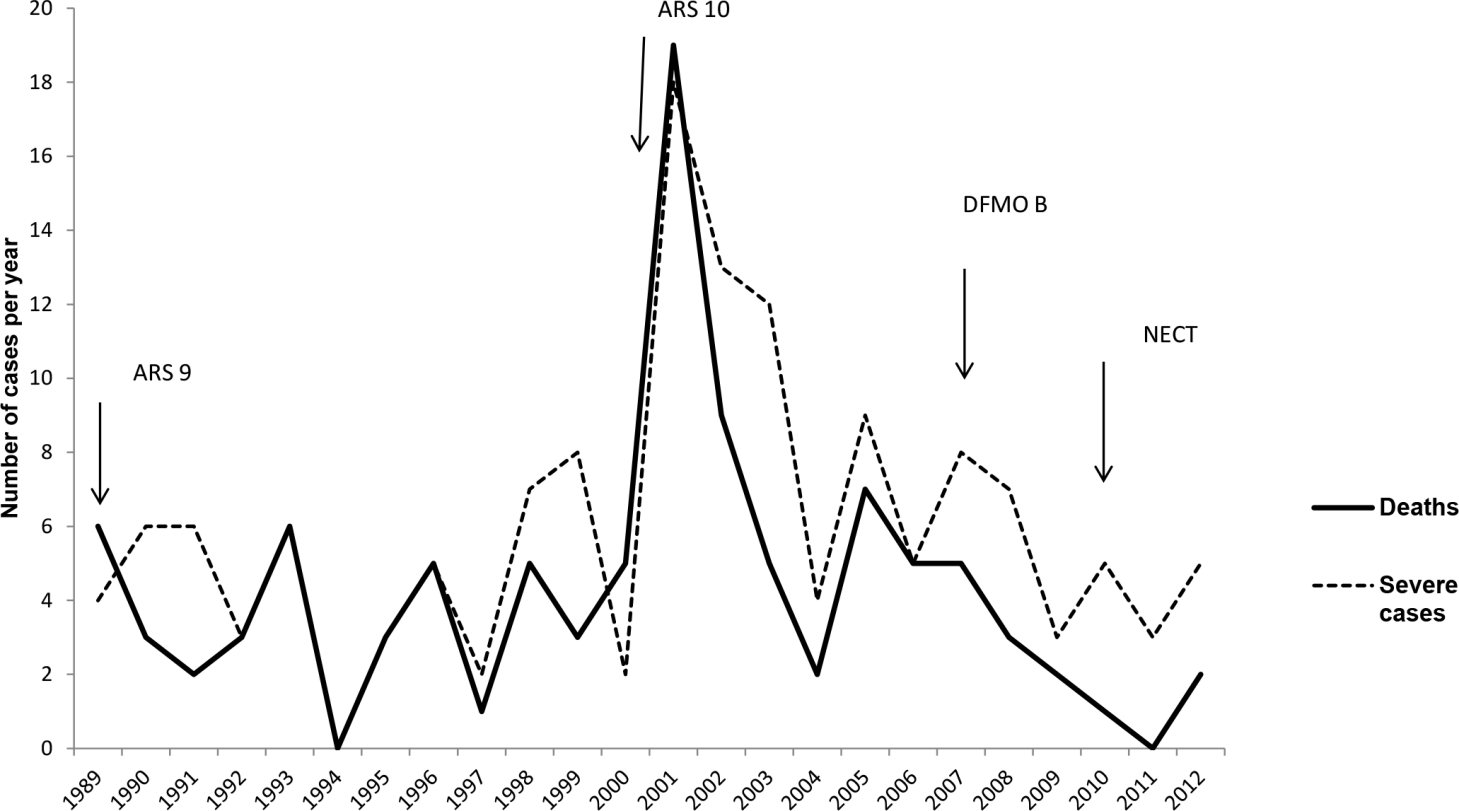
Yearly distribution of deaths and severely affected subjects on admission over the study period. Plain line = number of deaths per year; dotted line = number of severe (dysfunctional) cases on admission per year.

**Fig 3:**
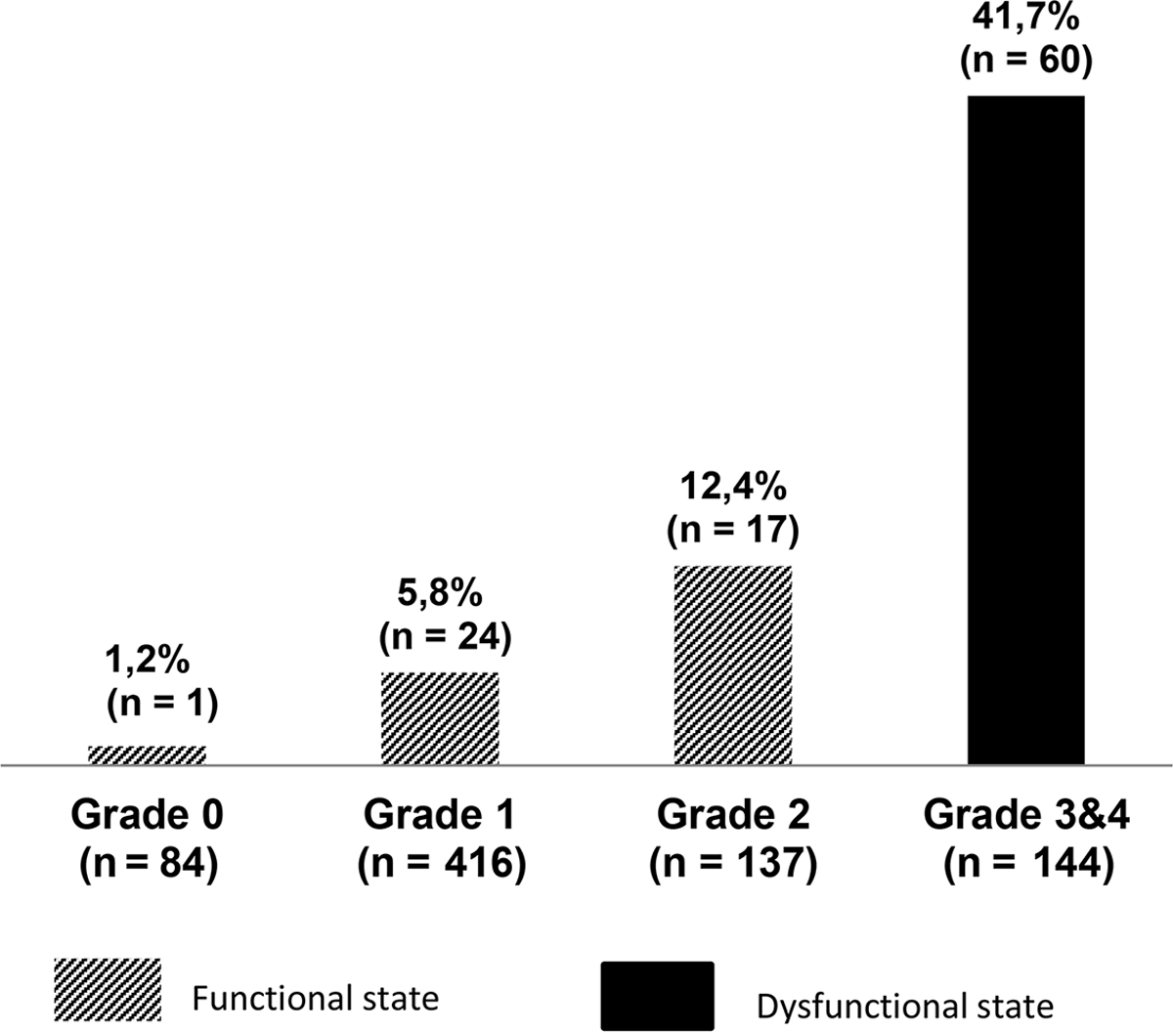
Proportion of deaths by severity grade on admission. N = number of patients admitted in one severity grade group; n = number of deaths in the corresponding severity grade group. Bars = percentage of deaths in each grade group. Black bars and dashed bars represent deceased patients among those admitted in dysfunctional (severely affected) and non-dysfunctional states, respectively.

Of the 781 patients included in the present analysis, death was recorded in 102 (13.1%) cases. The annual distribution of fatality rates varied over the study period and overlapped with the distribution of the dysfunctional cases and no peak of deaths was observed in 2007 (year of introduction of DFMO B) as compared to 2001. These characteristics suggest an influence of both, clinical presentation and treatment regimen on case fatality rate (Fig 2). Besides, the number of deaths increased consistently with disease severity on admission. Those admitted in non-dysfunctional state (Grade 0, 1 or 2) were interpreted as “unexpected deaths” (Fig 3).

**Fig 4:**
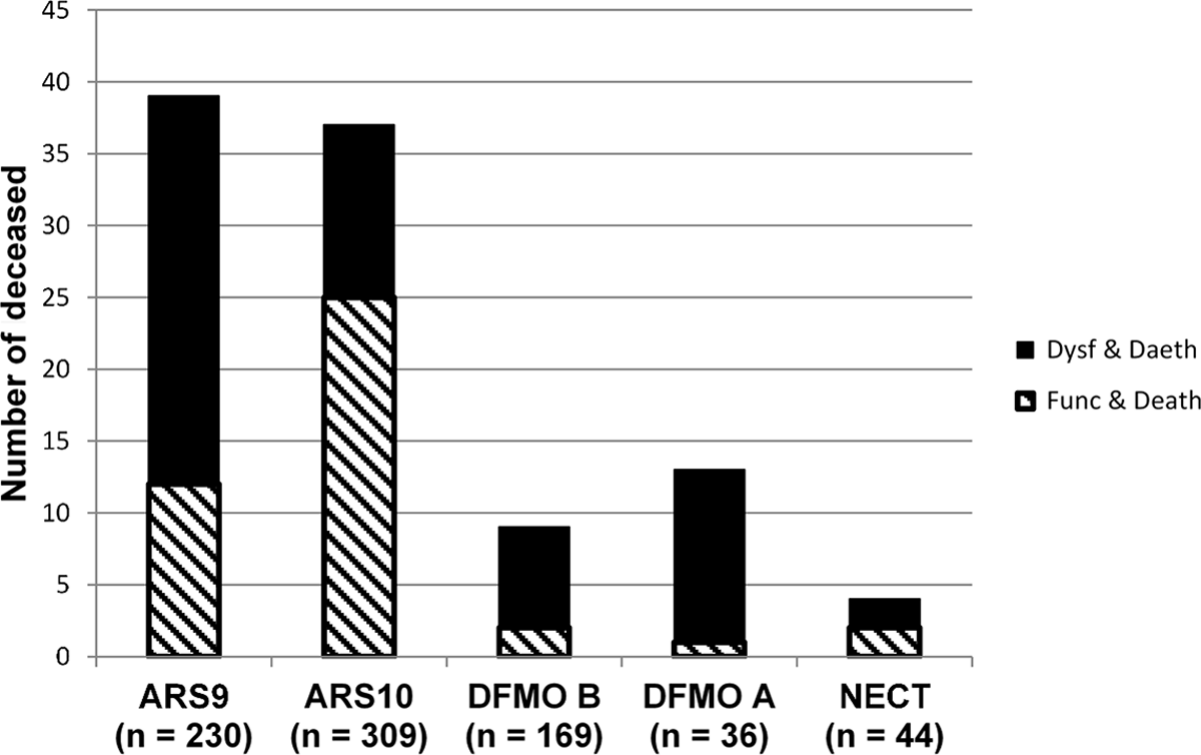
Proportion of deaths by clinical status on admission by treatment. Black bar = deceased, dysfunctional on admission; dashed bar = deceased, non-dysfunctional on admission.

Death rates per treatment group were as follows: 17.2% in the ARS 9 group, 12.1% in the ARS 10 group, 5.4% in the DFMO B group, 9.1% in the NECT group and exceptionally high (36%) in the DFMO A group. The difference in death rates across the five groups was statistically significant (Likelihood-ratio Chi2 (4) = 27.41; *p*<001). The difference in death rates between the two melarsoprol groups was not significant (Pearson Chi2 (1) = 2.8; *p* = 0.08). However, of the 42 deceased while clinically non-dysfunctional (not severely affected on admission), 37 (88.1%) were treated with melarsoprol. The highest proportion of non-dysfunctional cases among the deceased (“unexpected deaths”) was registered with ARS 10 (67.6%) in contrast to DFMO A (7.7%), which was administered exceptionally to a severely affected group (Fig 4). Differences in proportions of unexpected deaths were significant when comparing ARS 9 and ARS 10 (Pearson Chi2 (1) = 10.29; *p* = 0.001) or ARS 10 and DFMO B (Fisher exact test; *p* = 0.018)

Overall univariable regression analysis indicates that death was significantly associated with the clinical status on admission, presence of parasites in the CSF, treatment regimen, total protein concentration, and WBC in the CSF (Table 5).

**Table 5:** Unadjusted odds of death.

PREDICTORS	Crude OR (95% CI)	*P*
**Males**	1.19 (.79–1.81)	.403
**Age <15 years**	1.04.60–1.80)	.881
**Duration ≥6 months**	1.39.90–2.14)	.140
**Parasites in CSF**	2.04 (1.21–3.44)	.008
**Dysfunctional State**	10.12 (6.41–15.96)	.000
**CSF WBC**	1.00 (1.00–1.00)	.468
**CSF WBC ≥100 cells/ml**	1.70 (1.02–2.83)	.040
**CSF Proteins**	1.01 (1.00–1.01)	.000
**CSF Proteins ≥100 mg/dl**	2.03 (1.28–3.23)	.003
**Treatment**	1.27 (1.08–1.50)	.004
**Treatment Regimens**		
**DFMO B**	Reference	
**ARS 9**	2.09 (.70–6.17)	.184
**ARS 10**	1.37 (.46–4.05)	.569
**DFMO A**	5.65 (1.65–19.38)	.006
**NECT**	.57 (.17–1.93)	.364

Association models by treatment group showed that only dysfunction in 4 groups (ARS 9, ARS 10, DFMO A and DFMO B) and high total CSF protein concentration in 2 groups (ARS 10 and DFMO B) were significantly associated with death (Table 6).

**Table 6:** Unadjusted odds of deaths by treatment group.

Treatment	ARS 9, N = 226		ARS 10, N = 307		DFMO A, N = 36		DFMO B, N = 168		NECT, N = 44	
**Predictors**	Crude OR (95% CI)	*P*	Crude OR (95% CI)	*P*	Crude OR (95% CI)	*P*	Crude OR (95% CI)	*P*	Crude OR (95% CI)	*P*
**Males**	.77 (.37–1.57)	.46	1.96 (.96–4.01)	.07	0.37 (0.09–1.53)	.17	4.12 (0.83–20.47)	.08	0.45 (0.04–4.72)	.51
**Age <15 years**	.70 (.28–1.77)	.46	1.35 (.50–3.64)	.56	1.74 (0.44–6.97)	.43	2.51 (0.30–20.73)	.39	*	
**Duration ≥ 6 months**	1.42 (.70–2.88)	.33	1.39 (.68–2.84)	.37	0.69 (0.16–2.97)	.62	1.37 (0.27–7.00)	.71	1.00 (0.09–10.74)	1.00
**Parasites present in CSF**	1.58 (.65–3.81)	.31	1.94 (.85–4.40)	.12	0.50 (0.08–2.94)	.44	5.83 (0.71–47.70)	.10	*	
**Dysfunctional State**	17.79 (7.85–40.2)	.00	3.99 (1.81–8.78)	.00	11.00 (1.22–99.07)	.03	31.28 (6–163.57)	.00	5.67 (.66–48.33)	.11
**CSF WBC≥100 cells/ml**	1.25 (.58–2.68)	.56	1.38 (.62–3.06)	.43	*		4.35 (0.53–35.67)	.17	*	
**CSF Proteins ≥100 mg/dl**	1.23 (.59–2,56)	.57	2.24 (1.01–4.99)	.05	2.60 (0.52–13.04)	.25	8.74 (1.67–45.65)	.01	*	

*Not applicable due to limitations in sample size.

After adjusting for treatment, the following odds ratios were extracted: **10.40** [(95% CI: 6.55–16.51); *p* = .000] for clinical dysfunction (severely impaired initial clinical status), **2.14** [(95% CI: 1.35–3.39); *p* = .001] for high protidorachy, **1.99** [(95% CI: 1.18–3.37); *p* = .010] for the presence of parasites in the CSF and **1.70** [(95% CI: 1.03–2.81); *p* = .038] for high cytorachy; all in relation to death as the main outcome. A multivariable analysis within treatment groups retained clinical status on admission (in ARS 9, ARS 10 and DFMO B groups) and high protidorachy (in ARS 10 and DFMO B groups) as significant predictors of death (Table 7)

**Table 7:** Adjusted odds of deaths by treatment group.

Treatment	ARS 9 N = 226		ARS 10 N = 307		DFMO A N = 36		DFMO B N = 168		NECT N = 44	
**Predictors**	**OR** **(95% CI)**	**P**	**OR** **(95% CI)**	**P**	**OR** **(95% CI)**	**P**	**OR** **(95% CI)**	**P**	**OR** **(95% CI)**	**P**
**Males**	1.04 (.44–2.46)	0.92	2.16 (1.01–4.63)	0.05	.74 (.13–4.23)	0.73	6.24 (.77–50.80)	0.09	.14 (.004–4.73)	0.28
**Parasites present in CSF**	.87 (.26–2.91)	0.82	1.29 (.42–3;91)	0.66	.67 (.05–9.61)	0.77	1.13 (.08–16.44)	0.93	*	*
**Dysfunctional State**	19.26 (8.09–45.82)	0.00	3.31 (1.43–7.70)	0.00	27.74 (1.50–345.07)	0.24	16.09 (2.45–105.51)	0.00	15.24 (.85–271.59)	0.06
**CSF WBC≥100 cells/ml**	1.32 (.49–3.55)	0.58	.95 (.33–2.76)	0.93	*	*	1.61 (.13–20.61)	0.71	.12 (.00–3.18)	0.20
**CSF Proteins ≥100 mg/dl**	.76 (.31–1.87)	0.55	2.26 (1.01–5.07)	0.05	4.93 (.46–52.17)	0.18	9.33 (1.08–80.80)	0.04	*	*

## Discussion

This study explicitly discusses the role of clinical presentation on admission among current risk factors of death in a large series of second-stage HAT patients. Data from a 23 year-period of clinical management of HAT indicates that clinical presentation on admission, therapeutic regimen and protidorachy were main predictors of death, while high cytorachy was the weakest. One remarkable finding is the decrease in death rates at the advent of eflornithine as first-line treatment, a finding consistent with previous studies [5,25]. In general our study shows higher death rates than reported in HAT literature, as illustrated in the following studies. 17.2% versus 2.7–5% with the standard melarsoprol schedule (ARS9), [3,25,26], 12.1% versus 2.4–6.5% with the short melarsoprol schedule (ARS10), [5,25–27], 5.3% versus 0.4–3.1% with eflonithine (DFMOB) [5,25,28,29] and 9.1% versus 0.7–0.15% with NECT [30,31].The relatively higher rates in our series may be attributed to the so-called center effect for the admitting center is a tertiary-level unit devoted to neuropsychiatric care and serving as a reference center for the national program against HAT and, before 2001, most of the patients were admitted on the basis of neuropsychiatric manifestations. Patients admitted in such a setting are more likely to be diagnosed with advanced sleeping sickness[11,12]. The abnormally higher death rates in the group treated with eflonithine because of advanced disease (DFMOA: case fatality rate = 36%) is expected, as the best drug would not prevent the moribund from dying. The higher rate of death in the NECT group (9.1%) compared to first-line eflornithine (DFMO B) group (5.3%) in our series requires careful interpretation as NECT group had the highest rate of “dysfunctional” cases upon admission (18.2% versus 13.7%), and also higher frequency of cases with other indicators of disease severity (90.7% of cases with high CSF WBC count, 90.9% of cases with parasites in the CSF), compared to DFMOB group 66.1% and 59.5%, repectively) and the sample size was relatively small compared to other groups. In general recent studies show that NECT tends to perform even better than DFMO, at least it proved to be non-inferior in efficiency and of easier management [22] The low in-hospital case fatality rate registered with NECT by Alirol and al (0.15% or 1 of 684 second-stage HAT patients) has to be interpreted taking into account the low proportion (2.6%) of cases with high CSF cell count and a better general condition upon admission mostly in children.

Deaths occurring despite a relatively good clinical condition on admission were noted in 42 cases. One striking finding is that most of those unexpected deaths occurred with melarsoprol regimens. In 30 (80%) of the 37 treated with this drug, the severe adverse event that led to death was clearly an encephalopathic reaction; in 2 cases a non-encephalopathic reaction was observed with a preeminence of peripheral nervous system involvement that culminated with acute respiratory distress; in 1 case who died suddenly a cardiogenic etiology was suspected; in the 4 other cases the clinical picture was complex and difficult to interpret. At least in 86.5% of the cases (32/37) the severe adverse event could be related to the drug. Unexpected fatal outcomes raise the issue of the actual cause of death in treated HAT with the intricate question of drug toxicity [1–4,7,32], an open area for further research where, up to now, there are more questions than answers. Diverse situations can occur and a predictive algorithm like the one used in our study is essential in the process of assigning the probable cause of death to disease progression or to other causes such as drug toxicity or concurrent disease [2,7].

Among the biological factors CSF total proteins concentration prevailed in our study, a finding in line with the present day growing interest in proteomics to explore neuropathogenesis of HAT and improve disease staging [17,33–39] In fact, most articles dealing with disease severity are focused on biological stage determination as a basis for appropriate drug allocation [33,34,39,40]. The importance of clinical manifestations in the process of disease severity assessment is highlighted by the findings in a recent experimental animal study demonstrating that clinical events precede biological changes attesting neuroinvasion by trypanosomes [17]. Furthermore the authors point out to the need for an objective test to assess HAT clinical severity. The issue of stage determination is about to be settled with the advent of safe therapeutics efficient in both stages of HAT as stated above (“stage-independent oral drugs”) [20,22].

Several authors analyzed risk factors of death in treated HAT and some of them included clinical tests borrowed from pathological fields alien to HAT to assess disease severity such as Glasgow coma scale, Karnofski index. However the context of those studies was safety and effectiveness of therapeutic regimens and they did not explicitly discuss the role of clinical presentation among other risk factors of death [4,26,29,31] or they did not find a significant influence of clinical status on the outcome [4]. Literature tackling the issue of the influence of clinical presentation on the outcome of treated HAT is scarce. Two previous studies using a clinical index for disease severity to predict fatal outcome led to contradictory results [18]. In 1993 we published a paper showing a link between the number of neurological signs and fatal outcome in 2 groups of stage-2 HAT patients (19 children and 60 adults) treated with melarsoprol [19] The present study added a functional aspect to the clinical assessment procedure. This has allowed the correct assessment of the most affected subjects in whom elicitation of select neurological signs was compromised by lack of collaboration, in contrast to our former study where such patients were underrated. The merit of the present study is certainly the demonstration of the preeminence of the initial clinical status among factors influencing the outcome of treated stage-2 HAT. By properly categorizing 781 stage-2 HAT subjects, we demonstrated that patients admitted in severe clinical condition (“dysfunctional state”) were ~ 10 times more at risk of dying than those in relatively good clinical condition (“non-dysfunctional state”) on admission, irrespective of the treatment. Our protocol for the initial clinical assessment of subjects represents the first step towards the development of standardized and validated tools for a comprehensive case definition in sleeping sickness. Such tools would prove to be relevant in the clinical tailor-made management of HAT (e.g. to refer on an objective basis toward appropriately equipped settings for the care of the most affected patients), information exchange in epidemiological reports for disease surveillance and control, multicenter clinical trials and literature reviews. They would also provide objective grounds to circumstantiate imputability in pharmacovigilance.

## Supporting information

S1 Diagram Flow Admissions and selection of study subjects. St1, St2 = number of patients admitted in the first stage or second stage of HAT, respectively. Incomplete records = Stage-II HAT cases with incomplete clinical data.
(TIF)

S1 Checklist STROBE Checklist. (DOCX)
