# Supplementary figures and images for: Mortality trends and risk factors in advanced stage-2 Human African Trypanosomiasis: A critical appraisal of 23 years of experience in the Democratic Republic of Congo
Source: PLoS Negl Trop Dis. 2018 Jun 13;12(6):e0006504. doi: 10.1371/journal.pntd.0006504 (PMC5999091; doi:10.1371/journal.pntd.0006504)

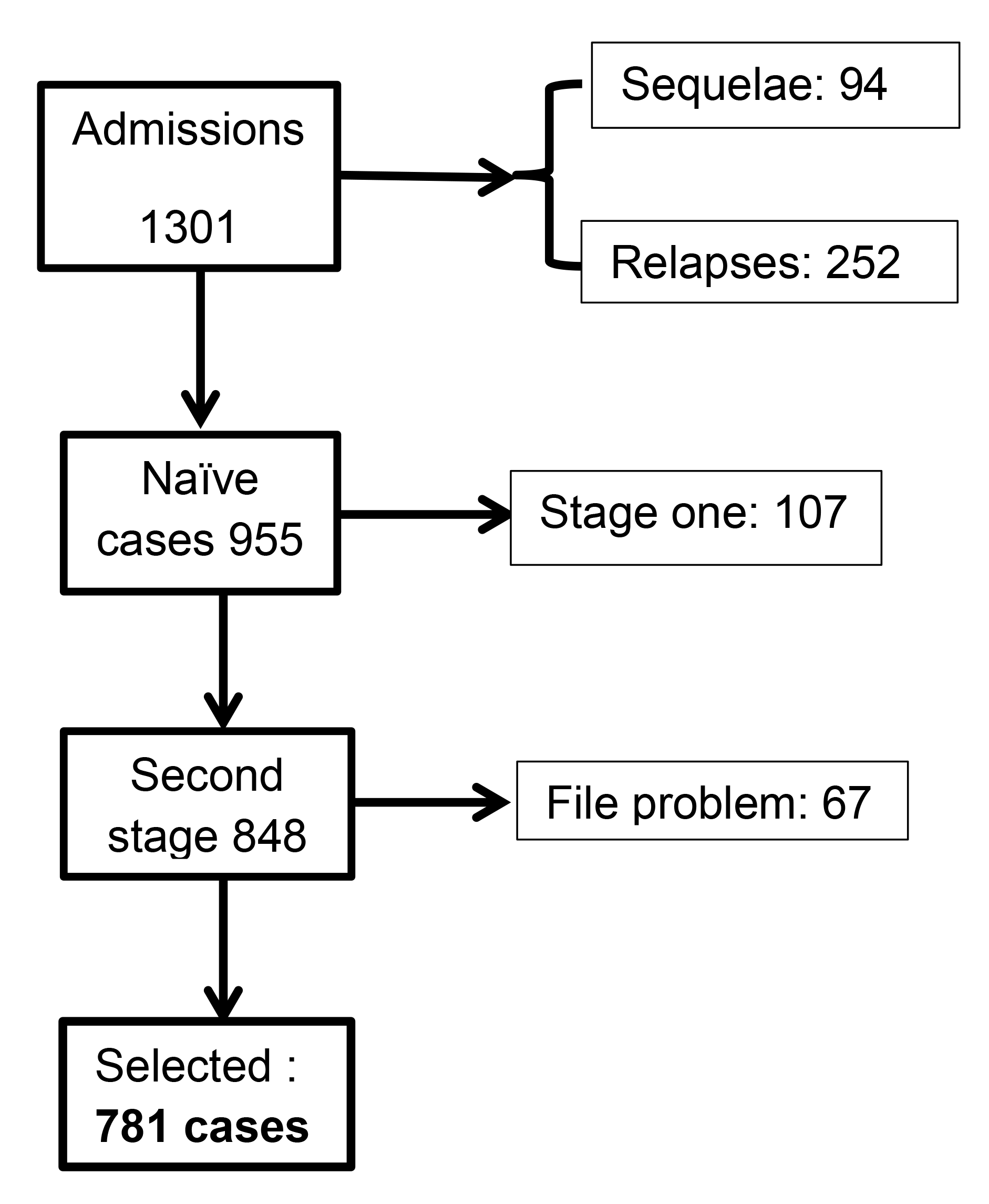

Supplement: S1 Diagram Flow — St1, St2 = number of patients admitted in the first stage or second stage of HAT, respectively. Incomplete records = Stage-II HAT cases with incomplete clinical data. (TIF) [file pntd.0006504.s001.tif]
