# Supplementary material for: Mortality trends and risk factors in advanced stage-2 Human African Trypanosomiasis: A critical appraisal of 23 years of experience in the Democratic Republic of Congo
Source: PLoS Negl Trop Dis. 2018 Jun 13;12(6):e0006504. doi: 10.1371/journal.pntd.0006504 (PMC5999091; doi:10.1371/journal.pntd.0006504)
Supplement: S1 Checklist — (DOCX) [file pntd.0006504.s002.docx]

STROBE Statement—checklist of items that should be included in reports of observational studies

|  | Item No. | Recommendation | Page  No. | Relevant text from manuscript |
| --- | --- | --- | --- | --- |
| **Title and abstract** | 1 | (*a*) Indicate the study’s design with a commonly used term in the title or the abstract | P 1, 2 | a retrospective study on mortality trends and risk factors |
|  |  | (*b*) Provide in the abstract an informative and balanced summary of what was done and what was found | P 2 | Death was the outcome variable whereas age, gender, duration of disease, location of trypanosomes in body fluids, cytorachy, protidorachy, clinical status (assessed on a syndromic and functional basis) on admission, and treatment regimen were predictors in logistic regression models  After adjusting for treatment, death odds ratios were as follows: **10.40** [(95% CI: 6.55-16.51); p = .000] for clinical dysfunction (severely impaired clinical status) on admission, **2.14** [(95% CI: 1.35-3.39); p = .001] for high protidorachy, **1.99** [(95% CI: 1.18-3.37); p= .010] for the presence of parasites in the CSF and **1.70** [(95% CI: 1.03-2.81); p = .038] for high cytorachy. |
| Introduction | | | |  |
| Background/rationale | 2 | Explain the scientific background and rationale for the investigation being reported | P 5 | In the process of assigning the probable cause of death to disease progression, treatment side effects, or any other factors including but not limited to coexistent pathology, the assessment of clinical status on admission among the baseline characteristics may be crucial  only few studies have addressed the issue of the prognostic value of clinical presentation in treated HAT |
| Objectives | 3 | State specific objectives, including any prespecified hypotheses | P 6 | intends to assess the impact of pre-treatment clinical status as well as select biological factors on mortality rate. |
| Methods | | | |  |
| Study design | 4 | Present key elements of study design early in the paper | P 6 | We retrospectively examined hospital records of naïve cases of stage-II HAT |
| Setting | 5 | Describe the setting, locations, and relevant dates, including periods of recruitment, exposure, follow-up, and data collection | P 6 | in the Unit of HAT of the Department of Neurology at the University of Kinshasa from 1989 to 2012 |
| Participants | 6 | (*a*) *Cohort study*—Give the eligibility criteria, and the sources and methods of selection of participants. Describe methods of follow-up  *Case-control study*—Give the eligibility criteria, and the sources and methods of case ascertainment and control selection. Give the rationale for the choice of cases and controls  *Cross-sectional study*—Give the eligibility criteria, and the sources and methods of selection of participants | P 7 | Subjects with confirmed parasitological diagnosis of HAT… stage-2 disease, and complete medical records were included |
|  |  | (*b*) *Cohort study*—For matched studies, give matching criteria and number of exposed and unexposed  *Case-control study*—For matched studies, give matching criteria and the number of controls per case | NA |  |
| Variables | 7 | Clearly define all outcomes, exposures, predictors, potential confounders, and effect modifiers. Give diagnostic criteria, if applicable | P 9 | Death was considered as the dependent variable. Independent variables (predictors) included age, sex, duration of the disease, location of the parasites in body fluids at the time of the diagnosis, CSF WBC, CSF total protein concentration, clinical status on admission, and treatment regimen. |
| Data sources/ measurement | 8* | For each variable of interest, give sources of data and details of methods of assessment (measurement). Describe comparability of assessment methods if there is more than one group | P 9, 10 | The clinical status on admission was categorized following a bi-axial clinical assessment inventory Table 1 Severity Assessment Chart |
| Bias | 9 | Describe any efforts to address potential sources of bias | P |  |
| Study size | 10 | Explain how the study size was arrived at | P 7, 8 | Diagram Flow |

Continued on next page

| Quantitative variables | 11 | Explain how quantitative variables were handled in the analyses. If applicable, describe which groupings were chosen and why | P 10, 11 | Data on age, duration of disease, WBC, and total protein concentration in the CSF were dichotomized using arbitrary cut-off points of 15 years, 6 months, 100 WBC/ml, and 100 mg protein/dl, respectively. Clinical status on admission was dichotomized into “dysfunctional status” and “non-dysfunctional status” and parasitological diagnosis was analyzed considering the presence of trypanosomes whether in CSF or not. Treatment regimens were compared in reference to DFMO B regimen. |
| --- | --- | --- | --- | --- |
| Statistical methods | 12 | (*a*) Describe all statistical methods, including those used to control for confounding | P 11 | Logistic regression models were used to assess the association between death and the aforementioned independent variables including gender. Statistical analyses were conducted using Stata software (version 11.2, Stata Corp Inc.) at the significance level of 0.05. |
|  |  | (*b*) Describe any methods used to examine subgroups and interactions |  |  |
|  |  | (*c*) Explain how missing data were addressed | P 7 | Not included in the analyses |
|  |  | (*d*) *Cohort study*—If applicable, explain how loss to follow-up was addressed  *Case-control study*—If applicable, explain how matching of cases and controls was addressed  *Cross-sectional study*—If applicable, describe analytical methods taking account of sampling strategy | NA |  |
|  |  | (*e*) Describe any sensitivity analyses | NA |  |
| Results | | | | |
| Participants | 13* | (a) Report numbers of individuals at each stage of study—eg numbers potentially eligible, examined for eligibility, confirmed eligible, included in the study, completing follow-up, and analysed | P 7, 8 |  |
|  |  | (b) Give reasons for non-participation at each stage | P 7, 8 |  |
|  |  | (c) Consider use of a flow diagram | P 8 | Diagram Flow. Overview of Admissions and Selection of Study Subjects |
| Descriptive data | 14* | (a) Give characteristics of study participants (eg demographic, clinical, social) and information on exposures and potential confounders | P 13, 14, 15 | Table3.Socio-demographic, Biological and Clinical Characteristics by Treatment Group. |
|  |  | (b) Indicate number of participants with missing data for each variable of interest | P 15 | Table 3 |
|  |  | (c) *Cohort study*—Summarise follow-up time (eg, average and total amount) | NA |  |
| Outcome data | 15* | *Cohort study*—Report numbers of outcome events or summary measures over time | NA |  |
|  |  | *Case-control study—*Report numbers in each exposure category, or summary measures of exposure | NA |  |
|  |  | *Cross-sectional study—*Report numbers of outcome events or summary measures | P 16 | Of the 781 patients included in the present analysis, death was recorded in 102 (13.1%) cases. |
| Main results | 16 | (*a*) Give unadjusted estimates and, if applicable, confounder-adjusted estimates and their precision (eg, 95% confidence interval). Make clear which confounders were adjusted for and why they were included | P 18 | Table 4a. Unadjusted Odds of Death. |
|  |  | (*b*) Report category boundaries when continuous variables were categorized | P 11 | Data on age, duration of disease, WBC, and total protein concentration in the CSF were dichotomized using arbitrary cut-off points of 15 years, 6 months, 100 WBC/ml, and 100 mg protein/dl, respectively. |
|  |  | (*c*) If relevant, consider translating estimates of relative risk into absolute risk for a meaningful time period | NA |  |

Continued on next page

| Other analyses | 17 | Report other analyses done—eg analyses of subgroups and interactions, and sensitivity analyses | P 16, 17 | Unexpected deaths by treatment |
| --- | --- | --- | --- | --- |
| Discussion | | | | |
| Key results | 18 | Summarise key results with reference to study objectives | P 21 | . Data from a 23 year-period of clinical management of HAT indicates that clinical presentation on admission, therapeutic regimen and protidorachy were main predictors of death, while high cytorachy was the weakest. |
| Limitations | 19 | Discuss limitations of the study, taking into account sources of potential bias or imprecision. Discuss both direction and magnitude of any potential bias | P 21 | In general our study shows higher death rates than reported in HAT literature...  may be attributed to the so-called center effect for the admitting center is a tertiary-level unit devoted to neuropsychiatric care… |
| Interpretation | 20 | Give a cautious overall interpretation of results considering objectives, limitations, multiplicity of analyses, results from similar studies, and other relevant evidence | P 24 | By properly categorizing 781 stage-II HAT subjects, we demonstrated that patients admitted in severe clinical condition (“dysfunctional state”) were ~ 10 times more at risk of dying than those in relatively good clinical condition (“non-dysfunctional state”) on admission, irrespective of the treatment |
| Generalisability | 21 | Discuss the generalisability (external validity) of the study results | P 24 | Our protocol for the initial clinical assessment of subjects represents the first step towards the development of standardized and validated tools for a comprehensive case definition in sleeping sickness… |
| Other information | |  | | |
| Funding | 22 | Give the source of funding and the role of the funders for the present study and, if applicable, for the original study on which the present article is based | P 24 | Acknowledgment. |

*Give information separately for cases and controls in case-control studies and, if applicable, for exposed and unexposed groups in cohort and cross-sectional studies.

**Note:** An Explanation and Elaboration article discusses each checklist item and gives methodological background and published examples of transparent reporting. The STROBE checklist is best used in conjunction with this article (freely available on the Web sites of PLoS Medicine at http://www.plosmedicine.org/, Annals of Internal Medicine at http://www.annals.org/, and Epidemiology at http://www.epidem.com/). Information on the STROBE Initiative is available at www.strobe-statement.org.
